# MALNUTRITION RISK AMONG OLDER AMERICANS ACT (OAA) NUTRITION PROGRAM PARTICIPANTS IN 2022 AND 2023

**DOI:** 10.1093/geroni/igae098.4022

**Published:** 2024-12-31

**Authors:** Jaime Gahche, Lydia McGrath, Laura Borth, Shirley Chao, Judy Simon, MaryBeth Arensberg, Johanna Dwyer

**Affiliations:** NIH Office of Dietary Supplements, Bethesda, Maryland, United States; Tufts University, Boston, Massachusetts, United States; National Association of Nutrition and Aging Services Programs, Washington DC, District of Columbia, United States; FoodPolicy Insights, Boston, Massachusetts, United States; National Association of Nutrition and Aging Services Programs, Washington DC, District of Columbia, United States; Abbott Nutrition Division of Abbott, Abbott Nutrition Division of Abbott, Ohio, United States; National Institutes of Health, Bethesda, Maryland, United States

## Abstract

Identifying older adults at risk for malnutrition is essential to combat poor outcomes associated with malnutrition. In 2022, the National Survey of Older Americans Act Participants (NSOAAP) included the Malnutrition Screening Tool (MST), a validated, self-report of unintentional weight loss and poor appetite. This provided the first opportunity to evaluate malnutrition risk (MR, defined as a score ≥2) consistently in a representative sample of Older Americans Act (OAA) program participants. In this cross-sectional analysis of 2022 (n=3531) and 2023 (n=4159) NSOAAP data, MR prevalence was estimated for total sample and within OAA programs included in NSOAAP (home-delivered meals (HDM), congregate meals (CM), homemaker (HM), case management (CS), transportation services (TR)). 2022 overall MR prevalence was 19.4%, with no significant differences between programs. 2023 overall MR prevalence remained fairly stable (17.2%) with no significant difference vs. 2022 (p< 0.0984). 2023 MR prevalence was 20.8% (HDM), 9.9% (CM), 26.6% (HM), 21.1% (CS), 17.7% (TR). 2023 MR prevalence among CM participants was significantly lower compared to MR risk in other OAA programs and also significantly lower compared to 2022 (9.9% vs. 16.7% p< 0.0195). Further research is needed to understand these trends, which may be partially affected by pandemic-related program changes. These data indicate a substantial proportion of OAA program participants were at MR in 2022—with very little change in 2023, except decreased prevalence in CM program participants. The findings highlight the continued need for including MR measures in the NSOAPP to evaluate program effectiveness and help reduce malnutrition in older adults.

